# Intraoperative outcomes of robotic surgery across multiple multimodal systems

**DOI:** 10.1007/s11701-025-03060-3

**Published:** 2026-01-19

**Authors:** Antonio Fioccola, Ilaria Angioni, Isabella Fratti, Alessandro Monte, Maria Chiara Sighinolfi, Bernardo Rocco, Paolo Pietro Bianchi, Davide Chiumello

**Affiliations:** 1 Department of Health Sciences, University of Milan, Milan, Italy; 2 Department of Anesthesia and Intensive Care, ASST Santi Paolo e Carlo, San Paolo University Hospital, Via Di Rudinì 9, Milan, Italy; 3 Department of Health Sciences, General Surgery Unit, University of Milan, Milan, Italy; 4 Department of Urology, Fondazione Policlinico Universitario A. Gemelli IRCCS, Rome, Italy; 5 Università Cattolica del Sacro Cuore, Rome, Italy; 6 Coordinated Research Center on Respiratory Failure, University of Milan, Milan, Italy

**Keywords:** Robotic surgery, Multi-modular systems, Anesthesia, Kidney function, Mechanical ventilation

## Abstract

**Background:**

The emergence of new multi-modular robotic surgical systems, such as Hugo RAS and Versius, introduces architectural and ergonomic variations compared with the established Da Vinci platform. While their surgical performance has been widely investigated, limited data exist regarding anesthesiological outcomes. This study aimed to compare intraoperative anesthetic parameters across three robotic platforms in a tertiary academic center.

**Methods:**

A retrospective observational analysis was conducted on 258 consecutive patients who underwent robotic abdominal, urological, or gynecological surgery between January 2024 and June 2025. Patients were stratified according to the robotic platform used—Da Vinci, Hugo RAS, or Versius. Intraoperative variables, including Trendelenburg angle, anesthesia duration, pneumoperitoneum time, urine output, and fluid balance, were compared among groups.

**Results:**

The Da Vinci system was used in 68.6% of cases, followed by Versius (15.9%) and Hugo RAS (15.5%). No major differences were observed in anesthesiological or postoperative outcomes across platforms, except for a higher intraoperative urine output with Versius in gynecological surgery. Minor variations included a steeper Trendelenburg position in colorectal surgeries performed with Da Vinci and shorter operative and pneumoperitoneum times with Versius in abdominal wall procedures.

**Conclusion:**

Despite structural and ergonomic differences, the Hugo RAS and Versius systems demonstrated anesthesiological safety and intraoperative performance comparable to the Da Vinci platform, supporting their safe integration into clinical practice.

**Supplementary Information:**

The online version contains supplementary material available at 10.1007/s11701-025-03060-3.

## Introduction

Over the last years, the utilization of robotic surgery for abdominal, pelvic, and urological procedures has continued to rise [1–4]. Furthermore, in several international guidelines for oncologic surgery, robotic surgery has been suggested as the first approach, due to the better perioperative and postoperative outcome [5, 6]. Over recent years, the use of robotic surgery for abdominal, pelvic, and urological procedures has steadily increased [7]. In 2000, the Da Vinci robotic platform (Intuitive Surgical, Sunnyvale, CA, USA) was introduced, featuring a high-definition camera system and flexible operative arms [8]. Successive generations of this system have been released, with the most recent version comprising four independent and identical robotic arms and a three-dimensional video system. Currently, the Da Vinci system remains the global benchmark in robotic surgery, with over two decades of clinical use [9]. It is deployed in approximately 42 countries, predominantly in Europe and North America, but its adoption remains limited in Africa, South America, and parts of Asia, largely due to its high purchase and maintenance costs [10].

In addition to the expiration of Da Vinci system patents and ongoing advancements in robotic technology, several new robotic platforms have been developed through the years, with independent robotic arms and a three dimension video system [11, 12]. This has intensified competition among manufacturers to improve both the performance and affordability of surgical systems [13]. These new robotic platforms have stimulated further innovation, with the aim of providing enhanced three-dimensional visualization and fully articulating robotic arms to improve surgical precision [11, 12]. Consequently, the adoption of new robotic platforms has accelerated, with several already incorporated into clinical practice [10, 13].

Some of these newer systems differ substantially from Da Vinci in terms of architecture and technological features [14]. The introduction of these modular robotic platforms has presented various technological and procedural challenges, prompting investigation into their clinical outcomes, implementation processes, and training requirements from the surgeon’s perspective. Particularly, the Hugo RAS (Medtronic, Minneapolis, MN, USA) and Versius (CMR, Cambridge, UK) platforms employ open consoles with high-definition three-dimensional vision. Moreover, they have modular, independently mobile units (“carts”) positioned around the patient, allowing easier movement in the operative room (OR) [14–17]. The open console design enhances the surgeon’s sense of freedom, improving situational awareness and communication with the surgical team, optimizing ergonomics and comfort [18, 19].

However, limited data are currently available regarding the clinical impact of these newer robotic systems. Intraoperative physiological variables — including parameters of mechanical ventilation, hemodynamic, fluid management, and renal function under general anesthesia — are key indicators of patient safety and surgical efficacy [20]. The distinct OR configurations required by multimodular systems may influence patient positioning and introduce unique anesthesiological considerations.

The concurrent availability of three novel robotic surgical platforms within a single institution provides a unique opportunity for direct comparison. The aim of this study was to assess the impact of the Hugo RAS and Versius systems compared with the single-console Da Vinci platform on intraoperative clinical outcomes in abdominal, urological, and gynecological surgeries.

## Materials and methods

### Study design and population

This is a retrospective observational study on consecutive patients undergoing robotic surgery at the “*Azienda Socio Sanitaria Territoriale* (ASST) *Santi Paolo and Carlo”* (Milan, Italy), an academic tertiary center for robotic surgery provided with Da Vinci, Hugo RAS and Versius systems. All patients with an elective indication to robotic abdominal surgery (colorectal surgery, cholecystectomies, abdominal wall surgery), robotic prostatectomy or robotic hysterectomy between January 2024 and June 2025 were enrolled. Exclusion criteria were: patients aged under 18 years old, pregnant women, American Society of Anesthesiologists (ASA) score 4 or 5, patients with chronic kidney disease (CKD), patients with heart failure (HF), patients with Body Mass Index (BMI) > 40 kg/m^2^, urgent/emergent procedures.

Individual surgical indications were grounded on current clinical guidelines. Patient’s allocation to a specific robotic platform was unplanned and based upon operating room and/or system availability. Three dedicated surgical robotic teams were involved: abdominal surgery team (PPB), urology (BR) and gynecology (GG). All procedures were managed by the same anesthesiological team (AF, DC).

The study was approved by the institutional review board “Comitato Etico Territoriale Lombardia 1” (protocol number CET 407–2024). Informed consent was obtained according to Italian regulations.

### Data collection

The following variables were collected on a prospectively maintained database for each patient: (1) pre-operative variables (age, gender, ASA score [21], BMI, Charlson Comorbidity Index (CCI) score [22], Assess Respiratory risk in Surgical patients in CATalogna (ARISCAT) score [23]); (2) intra-operative variables (total duration of general anesthesia, surgical time, pneumoperitoneum, pre-operative fasting, fluid loss due to perspiration, total fluids administered, urine output, estimated blood loss and net fluid balance); (3) post-operative variables: length of hospital stay. Data were gathered in a prospectively maintained database (Excel sheet) and collected by personnel independent from the surgical team.

### Clinical management

During the preoperative phase, patients were provided with one or more peripheral venous catheters or, when indicated, a central venous catheter, as well as an arterial catheter if deemed necessary by the anesthesiologist based on individual comorbidities. Antibiotic prophylaxis was administered in accordance with local protocols, consisting of cefazolin 2 g or clindamycin 600 mg in patients with β-lactam allergy.

General anesthesia was induced with an opioid (typically fentanyl, 1–2 µg/kg), an intravenous anesthetic agent (propofol, 1–2.5 mg/kg), and a depolarizing or non-depolarizing neuromuscular blocking agent (suxamethonium, 1 mg/kg, or rocuronium, 0.6 mg/kg, respectively). Orotracheal intubation was performed with a cuffed endotracheal tube using either direct or video laryngoscopy, according to the anesthesiologist’s judgment. Following induction, all patients received urinary catheterization.

Anesthesia maintenance was achieved either with propofol plus remifentanil (total intravenous anesthesia) or sevoflurane plus remifentanil (balanced anesthesia). Drug titration was guided by processed electroencephalography (pEEG), with a target Bispectral Index (BIS) of 20–60 [24]. Neuromuscular blockade was maintained with rocuronium administered as boluses or continuous infusion, aiming for a train-of-four (TOF) count of 0–1 throughout the intraoperative period [25].

At the end of surgery, emergence from anesthesia was facilitated by reversal of neuromuscular blockade, when necessary, to achieve a TOF count of 4 with a TOF ratio > 0.9.

### Sample size calculation

The primary outcome of the study is the urine output. Pre-experimental data from patients undergoing robotic surgery at our centre showed a mean urinary output of 0.8 mL/kg/h with a standard deviation (SD) of 0.4 mL/kg/h. Following AKI definition from KDIGO guidelines [26], assuming a clinically relevant difference between groups of 0.5 mL/kg/h (mean urine output 0.5 mL/kg/h in group 1 vs. 1.0 mL/kg/h in group 2), with a SD of 0.4 mL/kg/h in both groups, we calculated that a minimum of 7 patients per group would be required within each subpopulation to detect this difference with a two-sided α of 0.05 and a power of 80% (β = 0.20).

### Statistical analysis

Patients were divided in groups, according to the platform employed; Descriptive data were expressed as mean ± SD for data with normal distribution, median and interquartile range for data with non-normal distribution. Normality of distribution of continuous variables was assessed by Shapiro-Wilks test. Categorical variables were expressed as a number and percentage of the total (%). Student’s t-test or Wilcoxon-Mann-Whitney U test were employed to assess differences between parametric or non-parametric continuous variables amongst two groups, as appropriate. Two-way Analysis Of Variance (ANOVA) test was used to assess differences between continuous variables amongst three groups. Tukey’s honestly significant difference (HSD) test was used to perform post-hoc comparisons for variables in which the ANOVA test showed a statistically meaningful difference. Chi-square test was employed to establish differences between categorical variables. A p value < 0.05 was considered as statistically significant. All the statistical analyses were performed using RStudio (R Core Team (2022); R Foundation for Statistical Computing, Vienna, Austria).

## Results

### Overall study population

**Fig. 1:**
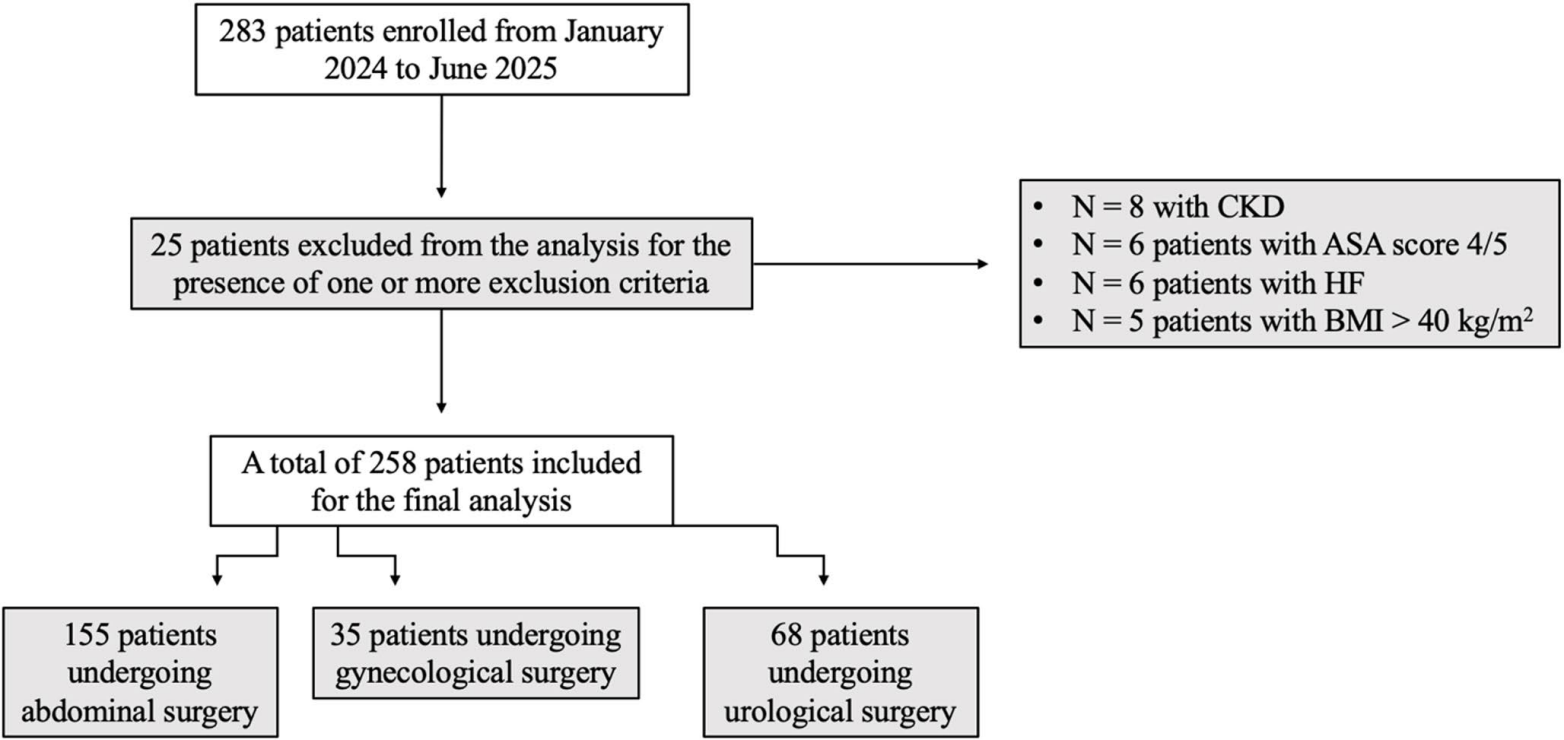
Patients’ enrolment flowchart. Legends: CKD, Chronic Kidney Disease; ASA, American Society of Anesthesiologists; HF, Heart Failure; BMI, Body Mass Index

Overall, 283 patients were enrolled in the study. As shown in Fig. 1, 25 patients were excluded from the analysis due to the presence of one or more exclusion criteria. Amongst these, 8 patients with CKD, 6 patients with an ASA score of 4 or 5, 6 patients with HF and 5 patients with a BMI over 40 kg/m^2^.

A total of 258 patients undergoing abdominal, gynecological or urological surgery were included in the analysis. In Table 1, surgeries performed stratified by robotic systems are shown. On the whole, Da Vinci was the commonest platform used to perform surgery (68.6% of the procedures), followed by Versius (15.9% of the procedures) and Hugo RAS (15.5% of the procedures) (Table 1).

**Table 1:** Population descriptive variables according to the robotic platform

	Da Vinci	Hugo RAS	Versius
**N (%)**	177 (68.6)	40 (15.5)	41 (15.9)
*Preoperative variables*			
**Female gender (N**,** %)**	71 (40.1)	20 (50.0)	33 (80.5)
**Age (years)**	63.9 ± 14.7	66.4 ± 13.7	56.0 ± 12.8
**ASA score**	2 [2–3]	2 [2–3]	2 [2–2]
**BMI (kg/m** ^**2**^ **)**	25.8 ± 5.4	26.2 ± 4.7	25.7 ± 4.4
**CCI**	4.0 ± 2.2	4.2 ± 2.4	2.1 ± 1.7
**ARISCAT score**	35.0 ± 11.9	34.6 ± 14.5	28.4 ± 11.2
*Intra-operative variables*			
**Total surgery time (min)**	241.0 ± 98.6	229.0 ± 82.0	199.0 ± 102.0
**Pneumoperitoneum (min)**	199.0 ± 85.8	186.0 ± 73.4	177.0 ± 103.0
**Trendelenburg (degrees)**	18.5 ± 9.9	14.6 ± 11.5	6.4 ± 9.2
**Total intravenous anesthesia (N**,** %)**	12 (6.7)	2 (5)	2 (4.8)
**Total fluids in (mL)**	1110.0 ± 83.9	2315 ± 698.0	1954.0 ± 816.0
**Urine output (mL)**	629.0 ± 47.5	320.0 ± 248.0	313.0 ± 308.0
**Urine output (mL/kg/h)**	1.56 ± 1.68	1.15 ± 0.91	1.60 ± 2.17
**Blood losses (mL)**	127 ± 9.6	151.0 ± 155.0	84.4 ± 76.6
**Fluid balance (mL)**	986.0 ± 74.5	−76.8 ± 652.0	−269.0 ± 655.0
**Tidal Volume (mL/kg)**	7.2 ± 1.3	7.2 ± 1.2	7.0 ± 1.1
**PEEP (cmH**_**2**_ **O)**	5 [5–5]	5 [5–5]	5 [5–5]
*Post-operative variables*			
**Length of hospital stay (days)**	6.5 ± 5.6	5.1 ± 5.5	3.0 ± 2.4

N, number; kg, kilograms; m, meters; min, minutes; mL, milliliters; kg, kilograms; h, hours; cmH_2_O, centimeters of water; ASA, American Society of Anesthesiologists; BMI, Body Mass Index, CCI, Charlson Comorbidity Index; ARISCAT, Assess Respiratory risk in Surgical patients in CATalogna. Post-hoc comparisons between statistically different variables amongst the three platforms are shown in Table S1

In Table 2, platforms were stratified for type of surgery performed. Da Vinci was the most employed platform in abdominal, urological and gynecological procedures (Table 2). Abdominal surgery procedures performed in our center include colorectal surgery, cholecystectomies and abdominal wall surgeries. Colorectal surgery was performed with all the three platforms, whilst abdominal wall repairs and cholecystectomy were performed with Da Vinci and Versius. Radical prostatectomies were performed with Da Vinci and Hugo RAS. Hysterectomies were performed with all the three platforms.    Overall, the Da Vinci system was predominantly used in urological surgery, which accounted for 70.6% of all procedures performed with this platform. In contrast, within the Hugo RAS caseload, urology represented the most frequent specialty (29.4%), followed by gynecology (25.7%), while abdominal surgery accounted for a smaller proportion of cases (7.1%). Notably, Da Vinci showed its highest utilization in abdominal surgery, with 107 patients treated, compared with 48 urological and 22 gynecological cases.

**Table 2:** Type of platform employed for type of surgery

Robot/Specialty	Abdominal surgery	Gynecological surgery	Urological surgery
Da Vinci (N, %)	107 (69.0)	22 (62.8)	48 (70.6)
Hugo RAS (N, %)	11 (7.1)	9 (25.7)	20 (29.4)
Versius (N, %)	37 (23.9)	4 (11.5)	0 (0.0)

N, number

### Pre-operative variables

Pre-operative and intra-operative variables stratified for the different surgeries are shown in Table 3 (colorectal surgery), Table 4 (gynecological surgery), Table 5 (urological surgery), Table S2 (cholecystectomies) and Table S3 (abdominal wall surgery). Pre-operative variables - stratified by intervention type - were similar across robotic systems.

### Intra-operative variables

#### Abdominal surgery: colorectal surgery

80 patients (79.2%) underwent colorectal surgery with the Da Vinci platform. The preoperative variables were not different among the three systems (Table 3). The Da Vinci system required a significantly steeper Trendelenburg position compared to the Hugo RAS (20.2 vs. 7.8, *p* = 0.0001) and Versius CMR (20.2 vs. 13, *p* = 0.02) (Table S4). The pneumoperitoneum duration and the amount of fluid administrated were not different amongst platforms. Further details about post-hoc comparisons are shown in Table S4.

**Table 3:** Robotic platform and outcomes/Colorectal surgery

	Da Vinci	Hugo RAS	Versius	*p*
**N (%)**	80 (79.2)	11 (10.9)	10 (9.9)	
*Pre-operative variables*				
**Female gender (N**,** %)**	32 (40.0)	9 (81.8)	9 (90.0)	0.06
**Age (years)**	68.2 ± 13.3	74.6 ± 14.1	63.6 ± 1.2	0.16
**BMI (kg/m** ^**2**^ **)**	25.0 ± 4.9	25.8 ± 5.9	24.9 ± 5.5	0.87
**ASA score**	2 [2–3]	3 [2–3]	2 [2–3]	0.59
**CCI**	4.51 ± 1.9	4.73 ± 2.4	3.5 ± 2.2	0.14
**ARISCAT score**	37.9 ± 11.6	47.6 ± 15.9	32.6 ± 8.9	0.07
*Intra-operative variables*				
**Total surgery time (min)**	306 ± 83.9	299 ± 66.5	324 ± 37.8	0.75
**Pneumoperitoneum (min)**	247 ± 82.3	248 ± 4	290 ± 34.2	0.24
**Trendelenburg (degrees)**	20.2 ± 7.4 *	7.8 ± 10.9 *	13 ± 6.1 *	**0.0001**
**Total intravenous anesthesia (N**,** %)**	12 (15)	2 (18)	2 (20)	0.25
**Total fluids in (mL)**	3235 ± 1080	2918 ± 578	2560 ± 804	0.11
**Urine output (mL)**	639 ± 833	432 ± 269	372 ± 322	0.44
**Urine output (mL/kg/h)**	1.78 ± 1.65	1.44 ± 0.97	1.12 ± 0.94	0.38
**Blood losses (mL)**	144 ± 111	200 ± 157	175 ± 75.5	0.26
**Fluid balance (mL)**	304 ± 104	410 ± 451	−276 ± 944	0.19
**Tidal Volume (mL/kg)**	7.50 ± 1.3	7.34 ± 1.2	7.35 ± 1.4	0.90
**PEEP (cmH**_**2**_ **O)**	5 [5–5]	5 [5–5]	5 [5–5]	0.90
*Post-operative variables*				
**Length of hospital stay (days)**	9.33 ± 6.6	6.82 ± 4.6	5.90 ± 1.1	0.14

N, number; BMI, Body Mass Index; ASA, American Society of Anesthesiologists; CCI, Charlson Comorbidity Index; ARISCAT, Assess Respiratory Risk in Surgical patients in CATalogna; min, minutes; mL, milliliters; kg, kilograms; h, hours; cmH_2_O, centimeters of water. *Multiple comparisons statistically significant (Tukey HSD): Hugo VS Da Vinci (*p* < 0.0001), Versius VS Da Vinci (*p* = 0.02). See Table S2 for details on the post-hoc test

#### Abdominal surgery: cholecystectomies

Cholecystectomies were performed with Da Vinci (42.9% of all the cholecystectomies) or Versius (57.1% of all the cholecystectomies) (Table S2). As shown, no difference regarding intra and post-operative variables has been highlighted.

#### Abdominal surgery: abdominal wall repair

Abdominal wall surgery was performed with either Da Vinci (54.6%) or Versius (45.4%). The pneumoperitoneum duration was significantly shorter with the Versius CMR compared to the Da Vinci system (68.8 min vs. 156 min, *p* = 0.03) (Table S3).

### Gynecological surgery

Table 4 shows the outcomes about gynecological surgery. In these patients, urine output differed between platforms, being higher with the Versius platform when compared to the Da Vinci (550 mL vs. 200 mL, *p* = 0.01) and to the Hugo RAS (550 mL vs. 150 mL, *p* = 0.002). However, as shown in Table 4, the Trendelenburg degrees employed (*p* = 0.96) and the duration of pneumoperitoneum (*p* = 0.66) were similar across the three platforms in gynecological surgery. Post-hoc comparisons are shown in table S5.

**Table 4:** Robotic platform and outcomes/Gynecological surgery

	Da Vinci	Hugo RAS	Versius	*p*
**N (%)**	22 (62.8)	9 (25.7)	4(11.5)	
*Pre-operative variables*				
**Female gender (N**,** %)**	22 (100.0)	9 (100.0)	4 (100.0)	0.99
**Age (years)**	48.3 ± 14.5	23.7 ± 54.1	52.6 ± 11.5	0.52
**BMI (kg/m** ^**2**^ **)**	28.6 ± 8.7	25.2 ± 4.9	24.7 ± 5.8	0.30
**ASA score**	2 [2–2]	1 [1–2]	1 [1–2]	0.08
**CCI**	2.0 ± 1.9	1.5 ± 0.7	1.6 ± 2.1	0.84
**ARISCAT score**	28.5 ± 16.1	23.6 ± 11.3	25.3 ± 12.2	0.68
*Intra-operative variables*				
**Total surgery time (min)**	161 ± 87.5	206 ± 9.10	148 ± 52.7	0.44
**Pneumoperitoneum (min)**	123 ± 71.3	176 ± 87	105 ± 65.5	0.66
**Trendelenburg (degrees)**	20.5 [20–25]	22 [20–24]	22 [20–25]	0.96
**Total intravenous anesthesia (N**,** %)**	0(0)	0(0)	0(0)	0.99
**Total fluids in (mL)**	1570 ± 471	2012 ± 920	1760 ± 744	0.28
**Urine output (mL)**	200 [100–500]*	150 [56.2–238]*	550 [500–825]*	**0.03**
**Urine output (mL/kg/h)**	0.98 [0.58–2.8]	0.90 [0.21–2.7]	3.58 [3.12–4.29]	**0.002**
**Blood losses (mL)**	98.8 ± 76.3	62.5 ± 58.2	90 ± 90.7	0.54
**Fluid balance (mL)**	−629 ± 760	−212 ± 632	−402 ± 677	0.36
**Tidal Volume (mL/kg)**	6.7 ± 1.4	7.9 ± 1.5	7.6 ± 1.1	0.07
**PEEP (cmH**_**2**_ **O)**	5 [5–5]	5 [5–5]	5 [5–5]	0.89
*Post-operative variables*				
**Length of hospital stay (days)**	3.6 ± 3.4	1.6 ± 1.4	2.1 ± 1.8	0.16

N, number; BMI, Body Mass Index; ASA, American Society of Anesthesiologists; CCI, Charlson Comorbidity Index; ARISCAT, Assess Respiratory Risk in Surgical patients in CATalogna; min, minutes; mL, milliliters; kg, kilograms; h, hours; cmH_2_O, centimeters of water. * Multiple comparisons statistically significant (Tukey HSD): Versius VS Da Vinci (*p* = 0.01), Versius VS Hugo (*p* = 0.008). See Table S5 for details on the post-hoc test.

### Urological sugery

Table 5 shows the pre-, intra and post-operative outcomes about urological surgery. Prostatectomies were performed with either Da Vinci (70.6% of all cases) or Hugo RAS (29.4% of all cases). No differences in intra-operative and post-operative variables were highlighted in these patients (Table 5).

**Table 5:** Robot platform and outcomes/Urological surgery

	Da Vinci	Hugo RAS	*p*
**N (%)**	48 (70.6)	20 (29.4)	
*Pre-operative variables*			
**Female gender (N**,** %)**	0 (0)	0 (0)	0.99
**Age (years)**	67.6 ± 6.4	68 ± 5.6	0.83
**BMI (kg/m** ^**2**^ **)**	25.7 ± 3.1	24.7 ± 2.3	0.25
**ASA score**	2 [2–2]	2 [2–2]	0.85
**CCI**	3.5 ± 1.7	3.9 ± 2.1	0.73
**ARISCAT score**	33.7 ± 9.0	36.5 ± 7.2	0.70
*Intra-operative variables*			
**Total surgery time (min)**	220 ± 71.6	230 ± 53	0.62
**Pneumoperitoneum (min)**	184 ± 62.9	185 ± 42.7	0.27
**Trendelenburg (degrees)**	24.7 ± 4.5	24.4 ± 1.6	0.72
**Total intravenous anesthesia (N**,** %)**	0(0)	0(0)	0.99
**Total fluids in (mL)**	2353 ± 765	2060 ± 401	0.11
**Urine output (mL)**	201 ± 250	290 ± 166	0.21
**Urine output (mL/kg/h)**	0.73 ± 0.99	1.10 ± 0.67	0.17
**Blood losses (mL)**	174 ± 167	240 ± 196	0.34
**Fluid balance (mL)**	−177 ± 817	−608 ± 434	0.46
**Tidal Volume (mL/kg)**	7.3 ± 1.1	7.1 ± 0.7	0.34
**PEEP (cmH**_**2**_ **O)**	5 [5–5]	5 [5–5]	0.92
*Post-operative variables*			
**Length of hospital stay (days)**	5.8 ± 3.0	5.7 ± 3.0	0.72

N, number; BMI, Body Mass Index; ASA, American Society of Anesthesiologists; CCI, Charlson Comorbidity Index; ARISCAT, Assess Respiratory Risk in Surgical patients in CATalogna; min, minutes; mL, milliliters; kg, kilograms; h, hours; cmH_2_O, centimeters of water.

**Fig. 2:**
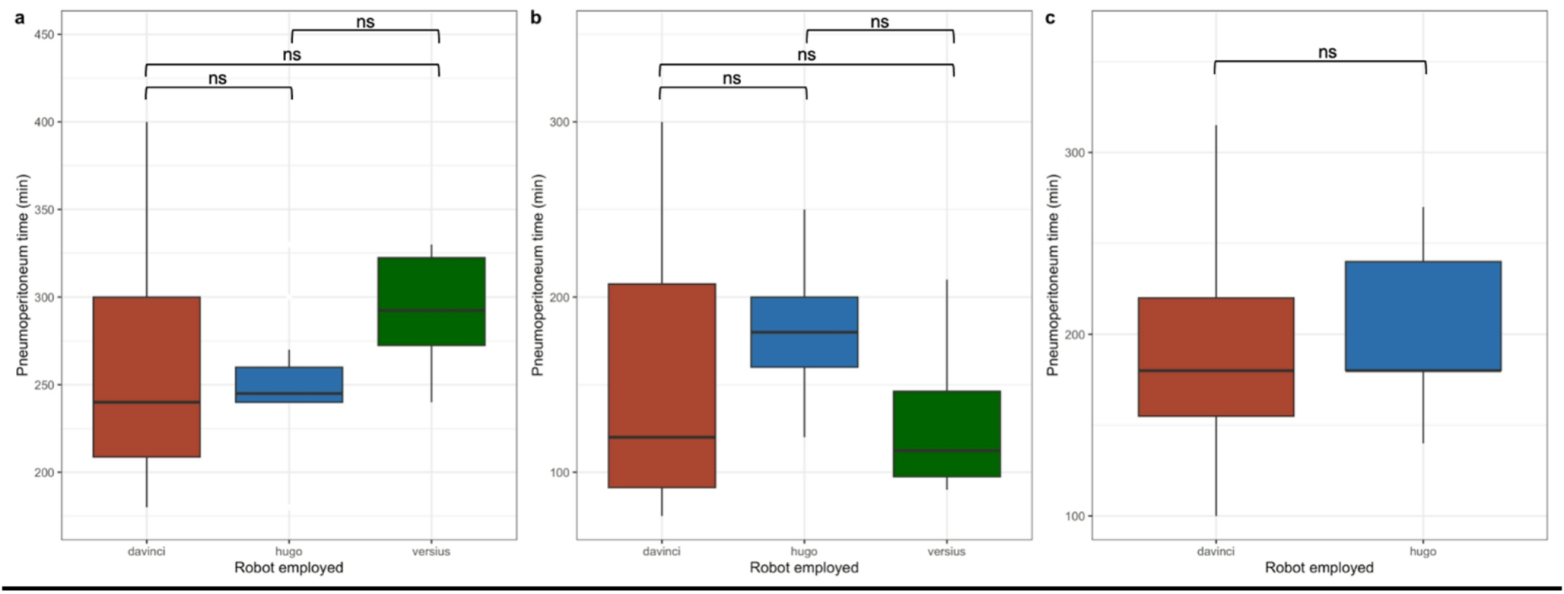
Pneumoperitoneum time with the different platforms used grouped per type of surgery. Panel a: abdominal surgery; panel b: gynecological surgery; panel c: urological surgery. Legends: ns, not statistically significant; min, minutes; davinci, Da Vinci platform; hugo, Hugo RAS platform; versius, Versius platform

As shown in Fig. 2, when grouped for the different surgery performed (colorectal, gynecological and urological surgery), pneumoperitoneum time was not statistically different across the different platforms.

## Discussion

### Major findings

Robotic surgery has achieved widespread adoption across multiple surgical specialties, including colorectal, urological, bariatric, upper gastrointestinal, and gynecological procedures. Since its introduction in the early 2000s, itshas become increasingly popular due to its recognized advantages in terms of safety, precision, and reproducibility. The Da Vinci Surgical System, the first commercially available platform, is now in its fifth generation. Analysis of over three million Da Vinci procedures has shown a malfunction rate below 1.0%, with conversion to open or laparoscopic surgery required in only 0.1% of cases [27].

Building on the Da Vinci’s success, several alternative systems have been developed worldwide [3, 7, 8], aiming to reduce costs and improve global accessibility. Among these, the modular Hugo RAS and Versius systems have drawn considerable attention, introducing distinct ergonomic and operational features that affect the entire surgical team [28].

In this study, we performed an intraoperative clinical comparison of the Da Vinci, Hugo RAS, and Versius systems. The Hugo RAS platform features an open console with pistol-grip controllers and four independent arm carts [15]. The Versius system, similarly modular, includes an open three-dimensional console requiring polarized glasses and handheld controllers, with four bedside units positioned around the patient [12]. Both systems demand optimized operating room (OR) layouts to avoid arm collisions and maintain surgical efficiency [13].

Preclinical data suggest that both platforms are intuitive and user-friendly [13, 23].

Given their modular design, these systems may influence intraoperative conditions through variations in OR configuration, patient positioning, docking, and instrument placement. Such factors could affect respiratory mechanics, hemodynamics, and fluid balance. Consequently, our study aimed to determine whether technological differences among robotic systems translate into anesthesiological outcome variations. Our findings indicate that all three platforms—Da Vinci, Hugo RAS, and Versius—are safe for abdominal, gynecological, and urological procedures, with only minor differences observed.

### Abdominal surgery

In colorectal procedures, the Da Vinci system required a steeper Trendelenburg position compared with Hugo RAS and Versius. However, this difference did not produce significant intraoperative or postoperative outcome variations.

Abdominal wall surgeries performed with Da Vinci and Versius revealed longer operative and pneumoperitoneum times with Da Vinci. This may reflect more complex case selection or differing setup efficiencies. Importantly, prolonged Da Vinci operating times were not associated with adverse intraoperative outcomes or longer hospital stays.

### Gynecological surgery

In hysterectomies with or without adnexectomy, patients operated with the Versius system exhibited higher intraoperative urine output than those with Da Vinci or Hugo RAS. The reason for this difference remains unclear, as variables influencing renal function—such as Trendelenburg angle and pneumoperitoneum duration—were comparable across groups. Nonetheless, urine output remained within physiological ranges (> 1 mL/kg/h) in all cases [29].

### Urological surgery

Our analysis of 68 robotic prostatectomies performed with Da Vinci and Hugo RAS showed no significant intraoperative differences, confirming comparable safety profiles. These results align with existing literature demonstrating equivalence between robotic platforms.

In one of the first single-center series comparing Da Vinci, Hugo RAS, and Versius for urological procedures, all surgeries were completed successfully without intraoperative complications [14]. Bravi et al. similarly found no significant differences between Da Vinci and Hugo RAS systems in prostatectomies regarding blood loss, complications, or operative time, although setup and docking were marginally longer with Hugo RAS [30]. Another small series reported a shorter mean operative time with Hugo RAS (153 ± 24 min vs. 165 ± 12 min) [31].

Three recent systematic reviews and meta-analyses corroborate these findings, demonstrating comparable safety and efficiency across platforms, with only minor docking delays observed for Hugo RAS [32–34]. Institutional co-adoption of Hugo RAS and Versius alongside Da Vinci has also proven feasible and safe, with no critical technical failures or clinically significant adverse events [35].

## Conclusions

Our findings suggest that, although multi-modular robotic systems may introduce theoretical challenges to surgical workflows, such challenges do not result in clinically significant impacts when appropriate planning and positioning protocols are implemented. Our results support the conclusion that, with experienced teams, the implementation of Hugo RAS and Versius systems does not require substantial modifications to perioperative protocols and maintains patient safety comparable to the Da Vinci system.

### Limitations

This study has several limitations. Its retrospective, non-randomized, and non-matched design introduces potential selection and information biases. Additionally, the predominance of Da Vinci procedures during the study period may have influenced the results. The Da Vinci platform remains the most established and widely used system, as confirmed by a recent meta-analysis [27], likely reflecting its sustained technological evolution and versatility across specialties. Another potential limitation of the present analysis is its focus on intraoperative data, only considering the length of hospital stay as postoperative outcome.

Despite these limitations, this study represents the first comparative analysis of anesthesiological parameters across multiple robotic platforms. As new systems from India, China, and Japan continue to emerge with varying designs, comprehensive multicenter evaluations are essential to inform evidence-based adoption and technological investment.

## Supplementary Information

Below is the link to the electronic supplementary material.

Supplementary Material 1

Supplementary Material 2

## Data Availability

All data supporting the findings of this study are available within the paper and its Supplementary Information.
