# Supplementary material for: Intraoperative outcomes of robotic surgery across multiple multimodal systems
Source: J Robot Surg. 2026 Jan 19;20(1):182. doi: 10.1007/s11701-025-03060-3 (PMC12816085; doi:10.1007/s11701-025-03060-3)
Supplement: Supplementary file 1 — Supplementary Material 1 [file 11701_2025_3060_MOESM1_ESM.docx]

**Intraoperative Outcomes of Robotic Surgery Across Multiple Multimodal Systems**

**Supplementary material**

**Table S1…………………………………………………………………….p.2**

**Table S2…………………………………………………………………….p.3**

**Table S3…………………………………………………………………….p.3**

**Table S4…………………………………………………………………….p.4**

**Table S5…………………………………………………………………….p.4**

Table S1. Post-hoc test (Tukey HSD) for continuous variables included in Table 1

| *Age (years)* | | | | |
| --- | --- | --- | --- | --- |
|  | **Difference** | **LWR** | **UPR** | **p** |
| **Hugo VS Da Vinci** | 1.86 | -4.01 | 7.73 | 0.74 |
| **Versius VS Da Vinci** | -7.87 | -13.3 | -2.47 | **0.002** |
| **Versius VS Hugo** | -9.73 | -16.9 | -2.58 | **0.004** |
| *ASA score* | | | | |
|  | **Difference** | **LWR** | **UPR** | **p** |
| **Hugo VS Da Vinci** | -0.15 | -0.41 | 0.11 | 0.38 |
| **Versius VS Da Vinci** | -0.30 | -0.55 | -0.06 | **0.009** |
| **Versius VS Hugo** | -9.73 | -0.47 | 0.16 | 0.49 |
| *CCI* | | | | |
|  | **Difference** | **LWR** | **UPR** | **p** |
| **Hugo VS Da Vinci** | 0.32 | -0.57 | 1.20 | 0.67 |
| **Versius VS Da Vinci** | -1.54 | -2.35 | -0.73 | **<0.0001** |
| **Versius VS Hugo** | -1.86 | -2.93 | -0.78 | **0.0002** |
| *ARISCAT score* | | | | |
|  | **Difference** | **LWR** | **UPR** | **p** |
| **Hugo VS Da Vinci** | 0.60 | -4.52 | 5.71 | 0.96 |
| **Versius VS Da Vinci** | -6.2 | -10.9 | -1.46 | **0.006** |
| **Versius VS Hugo** | -6.8 | -13.0 | -0.53 | **0.03** |
| *Total surgery time (min)* | | | | |
|  | **Difference** | **LWR** | **UPR** | **p** |
| **Hugo VS Da Vinci** | -11.8 | -52.3 | 28.7 | 0.78 |
| **Versius VS Da Vinci** | -41.6 | -78.9 | -4.37 | **0.02** |
| **Versius VS Hugo** | -29.8 | -79.1 | 19.5 | 0.33 |
| *Trendelenburg (degrees)* | | | | |
|  | **Difference** | **LWR** | **UPR** | **p** |
| **Hugo VS Da Vinci** | -3.9 | -8.1 | 0.3 | 0.07 |
| **Versius VS Da Vinci** | -12.1 | -15.9 | -8.2 | **<0.0001** |
| **Versius VS Hugo** | -8.2 | -13.3 | -3.1 | **<0.0001** |
| *Total fluids in (mL)* | | | | |
|  | **Difference** | **LWR** | **UPR** | **p** |
| **Hugo VS Da Vinci** | -250 | -672 | 171 | 0.34 |
| **Versius VS Da Vinci** | -612 | -999 | -224 | **0.0007** |
| **Versius VS Hugo** | -361 | -874 | 152 | 0.22 |
| *Blood losses (mL)* | | | | |
|  | **Difference** | **LWR** | **UPR** | **p** |
| **Hugo VS Da Vinci** | 15.5 | -36.3 | 67.4 | 0.76 |
| **Versius VS Da Vinci** | -51.4 | -99.1 | -3.7 | **0.03** |
| **Versius VS Hugo** | -66.9 | -130.0 | -3.8 | **0.03** |
| *Length of hospital stay (days)* | | | | |
|  | **Difference** | **LWR** | **UPR** | **p** |
| **Hugo VS Da Vinci** | -1.5 | -3.6 | 0.7 | 0.24 |
| **Versius VS Da Vinci** | -3.5 | -5.5 | -1.5 | **0.0001** |
| **Versius VS Hugo** | -2.1 | -4.6 | 0.6 | 0.17 |

Table S1. Post-hoc test (Tukey HSD) for continuous variables included in Table 1. Legends: LWR, lower bound of the confidence interval; UPR: upper bound of the confidence interval; mL, milliliters; kg, kilograms; h, hours; min, minutes; CCI, Charlson Comorbidity Index.

Table S2. Robotic platform and outcomes / **Cholecystectomies**

|  | **Da Vinci** | **Versius** | **p** |
| --- | --- | --- | --- |
| **N (%)** | 9 (42.9) | 12 (57.1) |  |
| *Pre-operative variables* | | | |
| **Female gender (N, %)** | 5 (55.6) | 7 (58.3) | 0.07 |
| **Age** | 60.8 ± 22.5 | 54.2 ± 14.4 | 0.45 |
| **BMI** | 23.9 ± 2.40 | 26.6 ± 4.22 | 0.09 |
| **ASA score** | 2 [2 - 2] | 2 [2 - 2] | 0.82 |
| **CCI** | 2.46 ± 2.38 | 1.95 ± 1.29 | 0.25 |
| **ARISCAT score** | 27.4 ± 12.9 | 22.7 ± 8.62 | 0.32 |
| *Intra-operative variables* | | | |
| **Total surgery time (min)** | 120 ± 76.4 | 93.7 ± 28.2 | 0.37 |
| **Pneumoperitoneum (minutes)** | 105 ± 27.4 | 68.8 ± 23.3 | 0.08 |
| **Trendelenburg (degrees)** | 0 [0 - 0] | 0 [0 - 0] | 0.98 |
| **Total fluids in (mL)** | 1256 ± 802 | 1225 ± 526 | 0.92 |
| **Urine output (mL)** | 16.7 ± 50 | 0 ± 0 | 0.35 |
| **Urine output (mL/kg/h)** | 0.14 ± 0.43 | 0 ± 0 | 0.35 |
| **Blood losses (mL)** | 30.6 ± 35 | 41.7 ± 35.9 | 0.48 |
| **Fluid balance (mL)** | -626 ± 1346 | -287 ± 564 | 0.49 |
| **Tidal Volume (mL/kg)** | 7.47 ± 1.17 | 6.71 ± 0.93 | 0.07 |
| **PEEP (cmH_2_O)** | 5 [5 - 5] | 5 [5 - 5] | 0.34 |
| *Post-operative variables* | | | |
| **Length of hospital stay (days)** | 3.60 ± 4.70 | 2.21 ± 2.91 | 0.42 |

Table S3. Robotic platform and outcomes/ Cholecystectomies. Legends: N, number; BMI, Body Mass Index; ASA, American Society of Anesthesiologists; CCI, Charlson Comorbidity Index; ARISCAT, Assess Respiratory Risk in Surgical patients in CATalogna; min, minutes; mL, milliliters; kg, kilograms; h, hours; cmH_2_O, centimeters of water.

Table S3. Robot type and outcomes / **Abdominal wall surgery**

|  | **Da Vinci** | **Versius** | **p** |
| --- | --- | --- | --- |
| **N (%)** | 18 (54.6) | 15 (45.4) |  |
| *Pre-operative variables* | | | |
| **Female gender (N, %)** | 12 (66.66) | 9 (60.00) | 0.97 |
| **Age** | 56.1 ± 12.3 | 54.1 ± 13.0 | 0.67 |
| **BMI** | 29.7 ± 7.30 | 25.9 ± 3.30 | 0.06 |
| **ASA score** | 2 [2 - 3] | 2 [2 - 2] | 0.07 |
| **CCI** | 2.13 ± 2.17 | 1.92 ± 1.44 | 0.76 |
| **ARISCAT score** | 31.5 ± 7.97 | 30.7 ± 12.4 | 0.83 |
| *Intra-operative variables* | | | |
| **Total surgery time (min)** | 181 ± 51.5 | 93.7 ± 28.2 | **0.04** |
| **Pneumoperitoneum (minutes)** | 156 ± 51.2 | 68.8 ± 23.3 | **0.03** |
| **Trendelenburg (degrees)** | 0 [0 - 0] | 0 [0 - 0] | 0.99 |
| **Total fluids in (mL)** | 1936 ± 557 | 1225 ± 526 | 0.19 |
| **Urine output (mL)** | 276 ± 165 | 112 ± 98 | 0.35 |
| **Urine output (mL/kg/h)** | 1.25 ± 0.73 | 0.76 ± 0.65 | 0.64 |
| **Blood losses (mL)** | 50 ± 52 | 41.7 ± 35.9 | 0.84 |
| **Fluid balance (mL)** | -291 ± 529 | -287 ± 564 | 0.51 |
| **Tidal Volume (mL/kg)** | 6.76 ± 1.24 | 6.93 ± 0.63 | 0.65 |
| **PEEP (cmH_2_O)** | 5 [5 - 7] | 5 [5 - 5] | 0.09 |
| *Post-operative variables* | | | |
| **Length of hospital stay (days)** | 2.47 ± 1.36 | 1.48 ± 2.31 | 0.75 |

Table S4. Robotic platform and outcomes/ Abdominal wall surgery. Legends: N, number; BMI, Body Mass Index; ASA, American Society of Anesthesiologists; CCI, Charlson Comorbidity Index; ARISCAT, Assess Respiratory Risk in Surgical patients in CATalogna; min, minutes; mL, milliliters; kg, kilograms; h, hours; cmH_2_O, centimeters of water.

Table S4. Post-hoc test (Tukey HSD) for Trendelenburg degrees with the three platforms / **Colorectal surgery**

|  | **Difference** | **LWR** | **UPR** | **p** |
| --- | --- | --- | --- | --- |
| **Hugo VS Da Vinci** | -12.40 | -18.40 | -6.45 | **< 0.0001** |
| **Versius VS Da Vinci** | -7.22 | -13.40 | -1.01 | **0.02** |
| **Versius VS Hugo** | 5.18 | -2.91 | 13.30 | 0.28 |

Table S2. Post-hoc test (Tukey HSD) for Trendelenburg degrees with the three platforms / Colorectal surgery. Legends: LWR, lower bound of the confidence interval; UPR: upper bound of the confidence interval; mL, milliliters; kg, kilograms; h, hours;

Table S5. Post-hoc test (Tukey HSD) for urinary output and urinary output per body weight per hour with the three platforms / **Gynecological surgery**

|  | **Difference** | **LWR** | **UPR** | **p** |
| --- | --- | --- | --- | --- |
| **Urine output (mL)** |  | | | |
| **Hugo VS Da Vinci** | - 80.8 | - 370.0 | 209.0 | 0.77 |
| **Versius VS Da Vinci** | 337.0 | 14.2 | 660.0 | **0.04** |
| **Versius VS Hugo** | 418.0 | 41.2 | 794.0 | **0.02** |
| **Urine output (mL/kg/h)** |  |  |  |  |
| **Hugo VS Da Vinci** | -0.58 | -2.65 | 1.48 | 0.90 |
| **Versius VS Da Vinci** | 2.53 | 0.54 | 4.53 | **0.01** |
| **Versius VS Hugo** | 3.12 | 0.71 | 5.52 | **0.008** |

Table S5. Post-hoc test (Tukey HSD) for urinary output and utinary output per body weight per hour with the three platforms / Hysterectomies +/- annexectomies. Legends: LWR, lower bound of the confidence interval; UPR: upper bound of the confidence interval; mL, milliliters.
